# Viewing Fantastical Events in Animated Television Shows: Immediate Effects on Chinese Preschoolers’ Executive Function

**DOI:** 10.3389/fpsyg.2020.583174

**Published:** 2020-12-11

**Authors:** Hui Li, Yeh Hsueh, Haoxue Yu, Katherine M. Kitzmann

**Affiliations:** ^1^Department of Preschool Education, Central China Normal University, Wuhan, China; ^2^Department of Counseling, Educational Psychology and Research, University of Memphis, Memphis, TN, United States; ^3^School of Psychology, Central China Normal University, Wuhan, China

**Keywords:** limited processing capacity, TV-EF, fNIRS, fantastical event, executive function, television, eye tracker

## Abstract

Three experiments were conducted to test whether watching an animated show with frequent fantastical events decreased Chinese preschoolers’ post-viewing executive function (EF), and to test possible mechanisms of this effect. In all three experiments, children were randomly assigned to watch a video with either frequent or infrequent fantastical events; their EF was immediately assessed after viewing, using behavioral measures of working memory, sustained attention, and cognitive flexibility. Parents completed a questionnaire to assess preschoolers’ hyperactivity level as a potential confounding variable. In Experiment 1 (*N* = 90), which also included a control group, there was an immediate negative effect of watching frequent fantastical events, as seen in lower scores on the behavioral EF tasks. In Experiment 2 (*N* = 20), eye tracking data showed more but shorter eye fixations in the high frequency group, suggesting a higher demand on cognitive resources; this group also did more poorly on behavioral measures of EF. In Experiment 3 (*N* = 20), functional near-infrared spectroscopy (fNIRS) data showed that the high frequency group had a higher concentration of oxygenated hemoglobin (Coxy-Hb), an indicator of higher brain activation consistent with a greater use of cognitive resources; this group also had lower scores on the behavioral EF tasks. The findings are discussed in reference to models of limited cognitive resources.

## Introduction

Executive function (EF) refers to the advanced cognitive processes (e.g., purposely switching attention) that regulate, control, and manage lower-level unconscious cognitive processes (e.g., automatic attention) when conducting complex cognitive tasks. The fundamental role of EF is to produce coordinated and purposeful behavior (Funahashi, 2001). EF develops rapidly across the preschool years (Carlson et al., 2012) and involves working memory, self-control, and flexible thinking, all of which undergird social (Eisenberg et al., 2004) and cognitive (Blair and Razza, 2007) function. EF in preschool also lays a foundation for the development of more advanced cognition (Garon et al., 2008) and success and well-being into adulthood (Mischel et al., 1989).

Researchers have long been interested in factors that disrupt preschoolers’ EF. One line of research has focused on the effects of viewing screen media in terms of duration, content and pace. The duration of young children’s screen viewing is a common concern among parents and pediatricians. Although the American Academy of Pediatrics (2016) has recommended that screen time be limited for young children, television and other screen media increasingly dominate young children’s daily lives. A survey study based on nearly 20,000 phone interviews with parents suggested that children ages 2 to 5 spent on average about 2 h each day using digital screens (Przybylski and Weinstein, 2019), half of which was devoted to non-interactive media like TV and video/DVD (Common Sense Media, 2017), also called receptive viewing (Anderson and Davidson, 2019).

It is worth noting that a related area of psychological research has also focused on children’s understanding of fantasy, in contexts other than receptive viewing. Several studies have shown that engaging in active fantasy-oriented play or fantastical pretense may directly enhance preschool children’s EF development (Thibodeau-Nielsen et al., 2016; Thibodeau-Nielsen et al., 2020). By turning toward EF, these studies extended earlier findings showing that magical or impossible events in film (Subbotsky et al., 2010; Richert and Schlesinger, 2016) and the exercise of imagination (Bunce et al., 2017) may facilitate preschoolers’ creative thinking. With this acknowledgment, we turn to the relations between screen time and EF in preschoolers.

### Screen Time and Preschoolers’ Executive Function

Does time spent watching non-interactive screen content affect preschoolers’ executive function? Previous evidence has not been consistent regarding the relation between children’s television or video exposure and their EF (for a review, see Kostyrka-Allchorne et al., 2017). Nathanson et al. (2014) found that preschool children who started watching television at an earlier age, and who had higher overall exposure to television, had lower scores on behavioral measures of EF. By contrast, Linebarger et al. (2014) found that exposure to entertainment television was associated with higher executive functioning among higher SES children, based on parent reports. Some researchers argue that receptive screen time like television viewing is an open field in which the relations between screen time and young children’s EF development merit extensive research (Yang et al., 2017).

One specific concern is about TV viewing duration in relation to preschoolers’ attention problems, which are broadly termed inattention (Kostyrka-Allchorne et al., 2017). Martin et al. (2012) examined the associations between “chaos” in the household (lack of routine, family instability, noise, crowding, and having the television on habitually) as predictors of poor attention. Only the variable for the family’s habitual TV use predicted preschoolers’ attention problems. High levels of television exposure have been shown to be associated with lower attention (Verlinden et al., 2012) and inattention/hyperactivity (Cheng et al., 2010; Ebenegger et al., 2012). Miller et al. (2007) found that television exposure was related to ADHD behaviors at school but not to parent reports of the child’s inattention at home (Miller et al., 2007). Other studies found no effects on hyperactivity (Conners-Burrow et al., 2011) or symptoms of ADHD (Stevens and Mulsow, 2006).

Researchers have long been aware that the question of whether there is a meaningful relationship between TV exposure duration and attention deficits or EF may depend on the TV content (e.g., Foster and Watkins, 2010). For example, Yang et al. (2017) reported the counter-intuitive finding that television viewing was associated with higher EF and they suggested that the association was mediated by exposure to child-directed educational programming. And yet, adult-directed programs have been shown to be associated with lower EF (Barr et al., 2010) and exposure to PG-13 and R rated movies has been found to be associated with hyperactivity (Conners-Burrow et al., 2011; Hsin et al., 2014). Disentangling the effects of duration and content is an important area of future research.

The fast pace that characterizes much of children’s programming may also affect preschoolers’ EF. Indeed, Wright et al. (1984) found that children who viewed a fast-paced program showed more gaze shifts and poorer memory of its content than children who viewed a slow-paced program. However, it has been a challenge for researchers to test pace independent of content. Geist and Gibson (2000) assigned children to watch the slow-paced *Mister Rogers’ Neighborhood* or the fast-paced *Mighty Morphin’ Power Rangers.* Children in the fast-paced group showed more inattention in a subsequent play session, but it is possible the finding was due to the content (educational vs. entertainment). To address this problem, Cooper et al. (2009) created videos that had the same content but differed in pacing. Children who watched the slow-paced video directed their attention more effectively in a subsequent performance task than those who watched the fast-paced video. Later, Lillard and colleagues (Lillard et al., 2015a,b) used a set of animation videos that varied in both content and pace to test whether a special type of content, i.e., fantastical events, was related to pacing. They found that the featured fantastical events, regardless of their pacing, disrupted subsequent EF.

In the current research we studied the immediate effects of viewing fantastical events on the EF of Chinese preschoolers. The words “animation” and “cartoon” are commonly translated into one Chinese expression, *donghua*. Thus, we will use the term animation throughout this paper. The children watched animations similar to those used in previous studies (Lillard and Peterson, 2011; Lillard et al., 2015b; Rhodes et al., 2020) but dubbed in Chinese. The choice to use dubbing was necessitated by the scarcity of options from the Chinese television and video market for children. Chinese programming for children relies heavily on United States importation (Tu, 2009). In addition, using United States videos provides a connection between earlier studies (e.g., Lillard and Peterson, 2011; Lillard et al., 2015b; Rhodes et al., 2020) and the current study. By using United States videos, the results of the current research can be interpreted within the frame of reference provided by the extant literature.

It is important to study samples from a non-Western population in this area of research in part because Chinese preschoolers have been reported to have higher scores on measures of inhibition and attention control (Lan et al., 2011) and other measures of EF (Sabbagh et al., 2006; Tan, 2020) than their United States counterparts. Chinese children’s EF is also thought to be heavily influenced by social interactions and relationships (Lewis et al., 2009; Tan, 2020). Thus, it is unclear whether viewing fantastical events would have the same immediate disruptive effect on Chinese preschoolers’ EF as it has been shown to have on the EF of children in the United States and United Kingdom.

### Adopting Promising Measures to Assess Executive Function

Behavioral measures are common in the field of children and media, and this type of assessment was used to test EF in all three experiments in the current study. However, there has been a call to adopt new methods and techniques for studying children in the process of viewing TV (Anderson and Hanson, 2009). One increasingly used technique is eye-tracking, which is a sensor technology that records information about a viewer’s gaze as a direct measure of visual attention and an indirect measure of information processing. Although this technology has been infrequently used to collect data on preschoolers’ visual attention while watching TV, it has been widely used in research on infants (Gredebäck et al., 2010), including infants’ visual search and attention to faces (Frank et al., 2014) and processing of video stimuli (Kirkorian et al., 2012). The eye tracker provides direct evidence of attention shifts (Hyönä, 2010; Kolling et al., 2014), with different lengths of eye fixation on various parts of the screen reflecting different underlying processes (Hawkins et al., 1997). As such, eye tracking data could be conducive to analyzing the mechanism by which EF is disrupted after viewing a given number of fantastical events on TV. This technology, in additional to behavioral measures, was used in Experiment 2 to assess EF.

Another method of assessment that can tap into the child viewer’s real time experience is the new technique of functional near-infrared spectroscopy (fNIRS), which can detect the successive changes in oxygenated hemoglobin known as Coxy-Hb a widely used index of changes in brain activation (Cui et al., 2011). The fNIRS technology is particularly well suited for preschool participants because the process has few body movement restrictions and is soundless (Moffitt et al., 2011). In the current study we used fNIRS to study neural activity in the prefrontal cortex (PFC), a location known as an important locus of EF. One recent study using fNIRS showed that when asked to judge the reality of fantastical events, 6-year-olds and adults showed similar accuracy, but the children took longer to decide and showed higher PFC activation, suggesting that they needed more cognitive resources for the task (Li et al., 2019). Experiment 3 in the current study used fNIRS, in addition to behavioral measures, to assess EF.

### Fantastical Events on the Screen and Limited Cognitive Processes

Woolley’s (1997) definition of fantastical events refers to physically unrealistic or impossible events. Even infants have the capacity to process some aspects of these events. Researchers studying privileged domains of knowledge posit that infants already possess representations of the laws governing physical events (Spelke, 1994; Goswami, 2008), and experimental studies have built a body of evidence to show that infants have a naïve theory of gravity (Shtulman and Carey, 2007; Morita et al., 2012; Frick et al., 2014). Presumably, this knowledge also helps infants recognize violations of physical laws, as occur in fantastical events. By preschool, children can give correct reality judgments of fantastical events (Li et al., 2015; Richert and Schlesinger, 2016).

Preschool children can process certain aspects of fantastical events as impossible; for example, they understand that a person cannot turn into a cup of coffee (Li et al., 2015). However, they still show confusion about animated educational content with entertaining fantasy (Mares and Sivakumar, 2014; Bonus and Mares, 2015) and have difficulty in cognitively reconciling the naïve physical theory from infancy with the so-called gravity error, i.e., the idea that a falling object can stay in the air (Bascandziev et al., 2016). This results in a short-term reduction in preschoolers’ EF after viewing fantastical events, because EF tasks and the processing of fantastical events rely on the same cognitive resources (Lillard et al., 2015b; Li et al., 2018). Even if they understand gravity, and understand that fantastical events are not real, preschoolers still have to process the gravity error and the story, potentially leading to cognitive overload. This is especially true because the media presentation can blend audio, socioemotional, linguistic, and/or narrative elements as parts of a fantastical event in the context of a story. Many entertaining television and video programs for preschoolers are replete with such cognitively taxing events.

The key assumption in the current study is that the excessive use of limited cognitive resources needed to process fantastical events reduces the availability of resources needed for executive function, just as posited by Kahneman (1973) in the influential book *Attention and Effort*. Kahneman held that there is one pool of attentional resources; if these resources are depleted by one task, then performance will suffer on another task. Fisch (2000) applied Kahneman’s concept to understanding the effect of limited working memory on children’s comprehension of educational TV content. He suggested that comprehending a narrative that is difficult or distanced from its education message will require additional processing resources, leaving fewer resources for other cognitive tasks such as processing the program’s educational content. Another elegant theory proposed by Lang (2000), called the limited cognitive capacity model, shares the same principles of the informational processing theory Fisch (2000) draws on.

The present research, involving three experiments, was designed to test whether viewing animated fantastical events on television would negatively affect EF in a sample of Chinese preschoolers. In the rest of the paper we will use the terms television, TV and video interchangeably, in all cases referring to non-interactive screens. The children in all three experiments were randomly assigned to watch an animation with a high number of fantastical events or to watch an animation with a low number of fantastical events. In Experiment 1, there was also a control group engaged in regular classroom activities. EF was tested immediately after the video (or comparable time in the classroom for the control group). The theoretical models of limited cognitive resources (Kahneman, 1973; Fisch, 2000; Lang, 2000) provided the conceptual framework for our assumption that viewing a video program with frequent fantastical events requires cognitive resources to direct attention, process information, and exercise self control, at the expense of cognitive resources needed for EF. We expected that the results would replicate earlier research but also identify potential physiological and neural mechanisms underlying the documented behavioral outcomes, constituting a unique contribution to the literature.

#### Hypotheses

Experiment 1 used a cognitive behavioral measure of EF to test the hypothesis that a TV program with more fantastical events would predict preschool viewers’ lower ability to keep and manipulate information in short term memory, lower inhibitory control over action, and lower cognitive flexibility. Experiment 2 used eye tracking data to test the hypothesis that there would be more but shorter fixations when preschoolers watched the video with more fantastical events, indicating lower sustained attention and greater cognitive load. Experiment 3 used fNIRS to test the hypothesis that the group viewing more fantastical events would show higher Coxy-Hb in PFC, an indicator of the use of neurocognitive resources and cognitive load.

## Experiment 1

Previous research in the United States and the United Kingdom documented that fantastical events on TV disrupted preschoolers’ and kindergarteners’ EF performance on behavioral tasks (Lillard and Peterson, 2011; Lillard et al., 2015b; Rhodes et al., 2020). Experiment 1 extended this research by examining whether these children’s Chinese counterparts also showed impaired EF immediately after viewing fantastical events on TV. EF was measured by established behavioral tasks that assess short term memory, sustained attention, and cognitive flexibility. We hypothesized that Chinese preschoolers who viewed a video with frequent fantastical events would show poorer performance on the behavioral EF tasks than those who viewed a video with infrequent fantastical events.

### Materials and Methods

#### Participants

Participants were 90 preschoolers (41 girls) between the ages 4 and 6 (M = 60.37 months, SD = 9.94 months, range = 48 – 78 months). The children attended an urban public preschool in central China. Most of their families had working- and middle-class annual household incomes (i.e., 4.4% below ¥36,000; 14.4% between ¥36,000 and ¥60,000; 77.8% above 60,000; 3.3% income data missing). They were randomly assigned to one of three conditions: Viewing a video episode with 46 fantastical events (high fantasy), viewing a video episode with 17 fantastical events (low fantasy), and no viewing (usual classroom activities). Each condition had 30 children, and the groups did not differ in gender (χ^2^(1, *N* = 90) = 1.94, *p* > 0.05) or age (χ^2^(1, *N* = 90) = 1.67, *p* > 0.05). Data collection took place at the children’s preschool. The Human Research Ethics Committee at the first author’s university approved the experiment’s materials and procedures. Parents provided written informed consent for their child to participate in the study.

#### Video Stimuli

Each video was presented on a 17-inch non-interactive laptop screen. The video in the low fantasy condition was the episode *Mickey’s Color Adventures* from the *Mickey Mouse Clubhouse* series (Gannaway, 2007) dubbed in Chinese, hereafter referred to as *Mickey*. This episode has a runtime of 19 minutes and 25 seconds. The video in the high fantasy condition was *Tom and Jerry*, hereafter referred to as *Tom*. Three episodes (*Flirty Birdy*, *the Cat Above and the Mouse Below*, and *Jerry’s Diary*) (Quimby et al., 1945, 1949; Jones et al., 1964) were shown in succession with a total runtime of 18 min and 37 s.

Research has shown that the immediate negative effect of viewing television on children’s EF is attributable to fantastical events, whether the events are fast- or slow-paced (Lillard et al., 2015a). To ensure that the pace of each video was coded and evaluated before the experiments, we first adopted an early definition: “Pace is actually composed of changes to a new scene (not previously shown in the program), changes to a familiar scene, and changes in the cast of characters present. These changes are often marked by visual features… or by auditory features…” (Wright et al., 1984, p. 654). Then, we identified scene changes by using *Scene Detector* (Scene Detector Pro, 2002), a computer program for automatically detecting video scene changes. There were on average 31.49 scenes per minute in *Mickey* and 29.40 in *Tom*, suggesting that the paces of the two videos were comparable. However, it is worth re-emphasizing that scene changes differ from the presence or absence of fantastical events.

Fantastical events were defined as physically impossible events (Woolley, 1997; Lillard and Peterson, 2011). For example, in *Tom*, Jerry floats slowly up in the air, and then floats slowly back down. Two graduate students separately coded each video for the number of fantastical events. *Mickey* showed 17 fantastical events and *Tom* showed 46 fantastical events. The overall between-rater agreement was 90.48% (57 out of 63). The disagreements on the remaining six fantastical events were resolved through discussion.

#### Procedure

In the two experimental groups, each child first spent 10 min with a trained research assistant in a waiting room to become acclimated to the setting, and then was escorted to the testing room that the researcher had set up in the preschool. The child was seated in front of a 17-inch non-interactive laptop screen. Once the child was ready, the experimenter said “Okay, we are about to watch an animation show. Please try to keep your head still while watching.” Children in the control group were monitored in their usual classroom activities for the same amount of time (18∼19 min). After the viewing session or the usual classroom session, the EF of each child was assessed using three behavioral tasks in a Latin Square design.

#### Measures

The three EF tasks consisted of the Backward Digit Span Task, the Day–Night Task, and the Flexible Item Selection Task. The raw scores for the three EF measures were highly correlated. The correlation coefficient between the Backward Digit Span Task and the Day-Night Task was 0.36 (*p* < 0.01); the correlation coefficient between the Day-Night Task and the Flexible Item Selection Task was 0.25 (*p* < 0.05); and the correlation coefficient between the Backward Digit Span Task and the Flexible Item Selection Task was 0.39 (*p* < 0.001). Thus, the child’s scores on all three EF tasks were converted to *z*-scores and summed to create a composite EF score.

The Backward Digit Span Task is a measure of working memory, specifically the ability to hold information in short-term memory and manipulate it. The task begins with a training phase in which the child is read four 2-number strings and asked to recite the string backwards (Lillard and Peterson, 2011). For example, if the experimenter said “2, 6,” then the child was expected to say “6, 2.” The experimenter gave feedback during training; when the child gave 1 correct answer, or after 4 training items (regardless of performance), the test trial was administered. The child was read up to 15 strings of randomly generated numbers ranging from 0 to 9, with string lengths beginning with 2 numbers and going up to 6. The experimenter stopped this task when the child did not give the correct answer on three successive test trials. The child received 1 point for each correctly recited string for a maximum of 15 points.

The Day-Night Task (Gerstadt et al., 1994) requires inhibitory control of action. The task uses a deck of 16 colorful cards, half of which depict the moon and stars, representing the nighttime, and the other half of which depict the sun and clouds, representing the daytime. The cards were randomly shuffled and then shown one by one to the child. However, the child was asked to say “night” to cards that depicted the sun and clouds, and “day” to cards showing the moon and stars. Each child was given 2 points for each correct answer (e.g., “night” for the sun and clouds card) and no points for wrong answers (e.g., “day” for the sun and clouds card). When the child gave a wrong answer at first but quickly corrected it, the child received 1 point. The sum of scores could range from 0 to 32.

**FIGURE 1:**
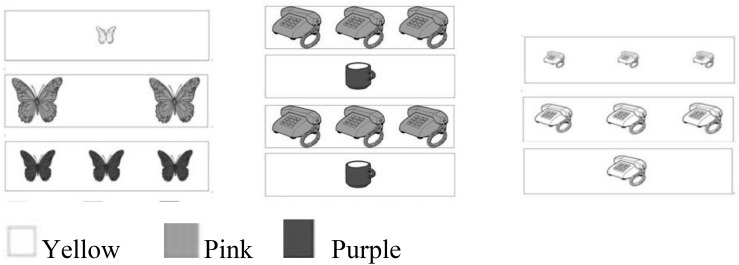
Examples of Demonstration (left), Criteria (center), and Test (right) trial cards from the Flexible Item Selection Task, adapted from a test of cognitive flexibility (Jacques and Zelazo, 2001).

The Flexible Item Selection Task was adapted from Jacques and Zelazo (2001) by substituting the socks and the fish with a cup and a butterfly that Chinese preschoolers could easily identify. This test of cognitive flexibility uses 48 white cards (28.5 × 7 cm), with one item on each card. These items could be described in one of four dimensions: Shape, color, number, and size (see Figure 1). The shape dimension refers to the structural features of the item, such as phone, butterfly, or cup. The color dimension refers to the color of the item such as pink, purple, or orange. The number dimension refers to how many items of different types appear on the card, such as 1, 2, or 3. The size dimension refers to whether the items were small (approximately 6.25 cm^2^), medium (approximately 20 cm^2^), or large (approximately 39 cm^2^).

The Flexible Item Selection Task consists of 15 trials: One demonstration trial, two criterial trials, and twelve test trials. The first three trials (one demonstration trial and two criterial trials) are always presented in the same order across all children. The experimenter uses the cards to introduce four dimensions (shape, color, number, and size) one by one during the demonstration trial (picture on left in Figure 1). The two criterial trials consist of a set of four cards. Two of them are identical to each other on all four dimensions (i.e., shape, color, number, and size), and the other two cards are also identical to each other on all four dimensions (picture at center in Figure 1). For example, two cards depicted three large pink phones and two cards depicted one medium purple cup.

The placement of matching pairs was counterbalanced across the above three trials. In the two criterial trials, the child was instructed to point at “two cards that are the same in one way” (Selection 1). When the child responded, the experimenter would ask the child to “choose two cards that are the same in another way” (Selection 2). The experimenter demonstrated the first criterial trial and made the first selection, “I will select the first card (from top to bottom) and the third card because they match each other in one way and I will select the second card and fourth card because they match each other in another way.” Once the child picked the correct cards and thus indicated their understanding of the task, the test trials started. Twelve sets of three cards were shown in the test trial. The experimenter asked the child to point at two cards that were the same in one way, using the same instructions as in the criterial trials, but from a set of three cards instead of four. The child did not receive any feedback in the selection process during the test trials. The child was given 1 point for each correct answer and no points for each incorrect answer or for giving no answer.

#### Questionnaire

Parents completed the Strengths and Difficulties Questionnaire (SDQ; Goodman, 2001) to provide information about their child’s hyperactivity as a potential confound in the study. We translated Goodman’s (2001) SDQ from English into Chinese for purposes of this study. The hyperactivity scale comprise 5 items: “restless, overactive, unable to stay still for long”; “constantly fidgeting or squirming”; “easily distracted, mind-wandering”; “thinking things out before acting”; and “inclined to persist in doing a task to the end, good attention span.” Parents rated each of the five items on a 3-point Likert scale: 1 = not true, 2 = somewhat true, or 3 = certainly true of the child. The last two items were reverse scored. The item scores were summed to create a hyperactivity score for each child, with lower scores indicating higher hyperactivity. Parents completed the questionnaire in the waiting room while their children participated in the experiment.

### Results

#### Preliminary Analyses

We first used one-way ANOVAs and chi-square tests to examine demographic variables across the three conditions: High fantasy, low fantasy, and control. The analyses indicated no significant group differences in demographics (sex, parents’ education, grandparenting, household income, and TV viewing time). The data from the hyperactivity subscale of the SDQ indicated that there were no significant group differences in hyperactivity (*M*_high fantasy_ = 3.87, *M*_low fantasy_ = 3.30, *M*_control_ = 4.17).

*Executive functioning*. A one-way ANOVA indicated a significant main effect of age (4, 5, 6 years old) on EF, *F*(2, 87) = 14.48, *p* < 0.001, η_p_^2^ = 0.25. *Post hoc* Tukey’s test results indicated 6-year-olds’ EF was higher than 4-year-olds’ EF, *t*(61) = 5.98, *p* < 0.001, Cohen’s *d* = 1.50 and 5-year-olds’ EF, *t*(56) = 2.94, *p* < 0.01, Cohen’s *d* = 0.80. There was no difference in the EF scores of 4- and 5-year-olds.

**FIGURE 2:**
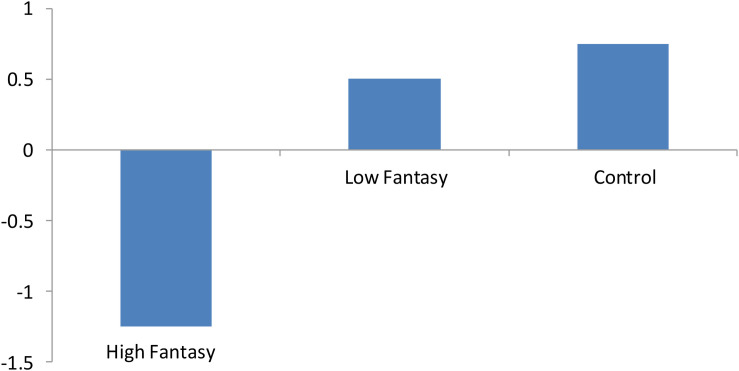
Summed *z* scores of three EF tasks by condition in Experiment 1. High Fantasy condition: Children watched *Tom*. Low Fantasy condition: Children watched *Mickey*. Control condition: Children engaged in regular classroom activities.

Based on these significant age effects on EF, an ANCOVA was used with age as the covariate to analyze whether there were main effects of condition on EF. The results showed that there was a significant main effect after controlling for age, *F*(2, 86) = 6.99, *p* < 0.005, η_p_ = 0.14 (see Figure 2). *Post hoc* Tukey’s tests indicated that after controlling for age, children in the high fantasy group had lower EF than children in the low fantasy group, *t*(58) = −2.56, *p* < 0.05, Cohen’s *d* = 0.66, and the control group, *t*(58) = −2.95, *p* < 0.01, Cohen’s *d* = 0.76. The latter two groups did not differ.

### Discussion

The results of Experiment 1 support the hypothesis that a higher frequency of fantastical events would predict lower EF, assessed with behavioral measures of the ability to keep information in short term memory and to manipulate it, inhibitory control over action, and cognitive flexibility. The findings of Experiment 1 add to the literature by providing evidence that Chinese preschoolers watching video programs with a high frequency of fantastical events also demonstrate the immediate post-viewing EF decline that has been documented among United States and United Kingdom preschoolers (Lillard and Peterson, 2011; Lillard et al., 2015a,b; Rhodes et al., 2020). In other words, on the behavioral level, Chinese preschoolers had EF disruption that was similar to what the Western samples experienced in a similar TV viewing condition.

Although earlier research showed that Chinese preschoolers had higher inhibition and attention control (Lan et al., 2011) and better performance on multiple EF measures (Sabbagh et al., 2006) than children in the United States, our data suggest that viewing frequent fantastical events disrupted EF in this Chinese sample. However, our data cannot address the possibility that Chinese children’s EF is influenced by social interactions and relationships, as was found in two studies (Lewis et al., 2009; Tan, 2020), because the experimental process was isolated and task-specific.

Based on Kahneman’s conceptual model we assumed that television with more fantastical events would lead to poorer EF task performance. Kahneman’s model would predict that frequent fantastical events in a TV program disrupt young children’s well-rehearsed representations from infancy by demanding more cognitive resources to be mobilized to grasp the events. In following this conceptual model, it may be reasoned that preschoolers’ understandings of the fantastical events were incongruent with the naïve theory of physics they had developed earlier. It was thus necessary for them to draw on extra cognitive resources in the viewing process, resulting in insufficient resources to achieve high scores on the behavioral measures of EF. Another challenge to preschoolers may arise from the presentation of media content, either in realistic or in anthropomorphic visuals. For example, animated or anthropomorphized science educational content can engender preschooler’s confusion even a week after the viewing session (Bonus and Mares, 2015; Bonus, 2019), which may suggest a form of burden on cognitive resources.

Although viewing videos of high vs. low fantastical events in this study demarcated the Chinese preschoolers’ performance on a cognitive behavioral EF task, the results do not provide direct evidence of cognitive overload as the mechanism that would explain the link between viewing fantastic events and lower EF. To establish cognitive overload as the mechanism, it would be important to ascertain whether preschoolers indeed tried to follow the events on the screen. This need to explain the link between fantastical events on TV and impaired EF motivated our next experiment.

## Experiment 2

In the second experiment we used eye tracking technology to measure Chinese preschoolers’ patterns of visual attention as a marker of cognitive load while viewing a video with either frequent or infrequent fantastical events. Researchers have used eye tracking measures to infer children’s cognitive load while children do various tasks. For example, eye tracking has been used to measure children’s cognitive processes in coding tasks (Papavlasopoulou et al., 2018), to identify children’s problem solving efforts (Wu et al., 2020), and to assess how children with high or low EF learn with a tablet device (McEwen and Dubé, 2015). Likewise, eye tracking data can reveal when, where and for how long preschoolers gaze at different parts of the screen, thus providing information about their cognitive efforts. This psychophysiological evidence of cognitive effort can be meaningfully linked to the findings from Experiment 1 and can add to our understanding of the demand on cognitive resources when preschoolers view fantastical events. Because frequent eye fixation shifts with short fixation durations are indicative of a high demand for cognitive resources, we hypothesized that the high fantasy group viewing *Tom* would have more frequent and shorter fixations than the low fantasy group viewing *Mickey.*

### Materials and Methods

Twenty-two preschoolers were recruited to participate in the experiment. However, two participants were excluded because only 70% of their eye tracking information was recorded due to the child’s behavior and technical problems. In the final group of 20 preschoolers (9 girls), the ages were between 4 and 6 (*M* = 63.94 months, *SD* = 10.38 months, range = 47 – 78 months). These children attended the same public preschool where Experiment 1 was conducted. The children were randomly assigned to the high fantasy group (*n* = 10) or the low fantasy group (*n* = 10). The study was approved by the Human Research Ethics Committee of the first author’s university. Parents provided written informed consent for their child to participate.

A relatively small sample was chosen because of the difficulty in collecting eye tracking data from young children. It is not uncommon to have very small samples of preschoolers in experiments using the eye tracker (e.g., Evans and Saint-Aubin, 2005; d’Ydewalle and De Bruycker, 2007). In the current study each child had to stay seated throughout the viewing session, a challenging task for young children. Technically, the eye tracker requires limited head and body movements to secure adequate data, but a fidgety child may disregard these limitations (Wass, 2016).

#### Eye Tracking Instrument

A Tobii T120 Eye Tracker was set up at the bottom of the 17-inch non-interactive laptop screen to send out infrared light and record children’s eye positions at 60 Hz, allowing an optimal accuracy of 0.5°. Each eye fixation in a 30 × 30 pixel area that exceeded 100 milliseconds in duration was counted as one fixation. The children could move their heads within a range of 44 cm (width) × 22 (height) cm. Tobii Studio 2.0 produced the eye movement information output that captured the viewer’s fixation pattern while viewing.

#### Treatment and Measures

The videos were the same as those used in Experiment 1. That is, *Tom* was shown to the high fantasy group and *Mickey* was shown to the low fantasy group. The battery of behavioral measures of EF tasks was also the same. Each child was asked to sit still and to watch the entire video in front of a 17-inch non-interactive laptop screen, with a Tobii T120 Eye Tracker attached to the bottom of the screen. Following this viewing session, the EF tasks were administered immediately in an adjacent quiet room. Parents completed the Strengths and Difficulties Questionnaire (SDQ) in the waiting room while their children participated in the experiment.

### Results

The baseline data from the hyperactivity subscale of the SDQ showed that there was no significant group differences in hyperactivity (*M*_high fantasy_ = 3.40, *M*_low fantasy_ = 3.87). However, the high fantasy group scored significantly lower than the low fantasy group on the behavioral EF tasks, *t*(16) = −2.51, *p <* 0.05, Cohen’s *d* = 0.31.

The eye tracker captured two types of data: Total number of fixations and average duration of fixation. The high fantasy group had on average significantly more fixations than the low fantasy group (Mean Difference = 377.25), *t*(16) = −3.68, *p <* 0.005, Cohen’s *d* = 1.72. The average duration of fixation in the high fantasy group was significantly shorter than in the low fantasy group (Mean Difference = −86.82), *t*(16) = 4.93, *p <* 0.001, Cohen’s *d* = 2.29.

### Discussion

The findings in Experiment 2 based on eye tracking data supported the hypothesis that preschoolers in the high fantasy group would have more frequent but shorter fixations than those in the low fantasy group while watching the video. This difference suggests that the children in the high fantasy group showed more orienting responses, thus more attention shifts through their physical eye moments. Kahneman’s (1973) extensive review of research on the relations between eye movements and cognitive efforts suggested that “the movements of the ‘mind’s eye’ are correlated with those of the physical eye” (p. 62). We infer that, in the high frequency group, the mobilization of the mind’s eye may be relatively overactivated in an effort to process the fantastical events. As a result, the high fantasy group did relatively poorly on the behavioral EF tasks after watching the video. In other words, there was a greater use of cognitive resources when watching *Tom*, presumably at the cost of cognitive resources needed for EF. This finding is consistent with models of limited cognitive capacity (Kahneman, 1973; Fisch, 2000; Lang, 2000).

Experiment 2 was limited by the lack of a control group. Nevertheless, the eye-tracking provide a plausible explanation of the immediate EF impairment seen in Experiment 1. The eye tracking data showed a direct association between the high fantasy occurences and the short but frequent fixations. Following Kahneman’s (1973) model, our question was whether there was other evidence that would support the results from Experiments 1 and 2 and bring us closer to identifying a mechanism by which high fantasy video content decreased preschooler’s EF. This question gave rise to Experiment 3, which used the newer technology of fNIRS to examine the neurological mechanism of diminished EF.

## Experiment 3

Kahneman’s (1973) model of limited capacity allows us to infer from the findings of the two previous experiments that viewing fantastical events overloads processing resources, thus disrupting behaviors associated with EF. Experiment 3 built on the first two experiments by testing the neurological underpinnings of this immediate disruptive effect. That is, the processing of fantastical events can be assessed not only by eye movements, but also by measuring cerebral blood flow to PFC, a key area involved in executive function (Abraham et al., 2008). Cerebral blood flow to this area can be used as an indicator of the use of cognitive resources. fNIRS is a recent technology that afforded us such evidence by revealing regional neural activation in PFC in Chinese preschoolers while watching fantastical events in *Tom* and *Mickey*.

### Materials and Methods

#### Participants

Thirty-four Chinese preschoolers (18 girls) aged 4 to 6 were recruited from an urban Children’s Learning Center, a weekend-only early education provider, in a metropolitan area of central China. The Center served mainly middle-class families, of which 14.7% had an annual household income between ¥36,000 and 60,000; 79.4% above 60,000; and 3% below 36,000 (with 2.9% missing). The reason for choosing a relatively small sample of preschoolers was similar to that described in Experiment 2, namely, the care and time needed to set up wire connections to use the fNIRS technology with each participant. We also faced the constraint of limited access to this new technology. However, after the initial randomization to the high and low fantasy groups, there was attrition due to children leaving their seats or being excused to go to the bathroom. As a result, ten preschoolers (9 girls) from each group (*N* = 20) completed the viewing session (*M* = 63.94 months, *SD* = 10.38 months, range = 47–78 months). Although this was a small sample, its size was common in studies using fNIRS with preschoolers (Tsujimoto et al., 2004; Moriguchi et al., 2009; Moriguchi and Hiraki, 2011; Li et al., 2019). The experiment’s materials and procedures were approved by the Human Research Ethics Committee of the first author’s university. Parents provided written informed consent for their child to participate in the study.

#### Measures

**FIGURE 3:**
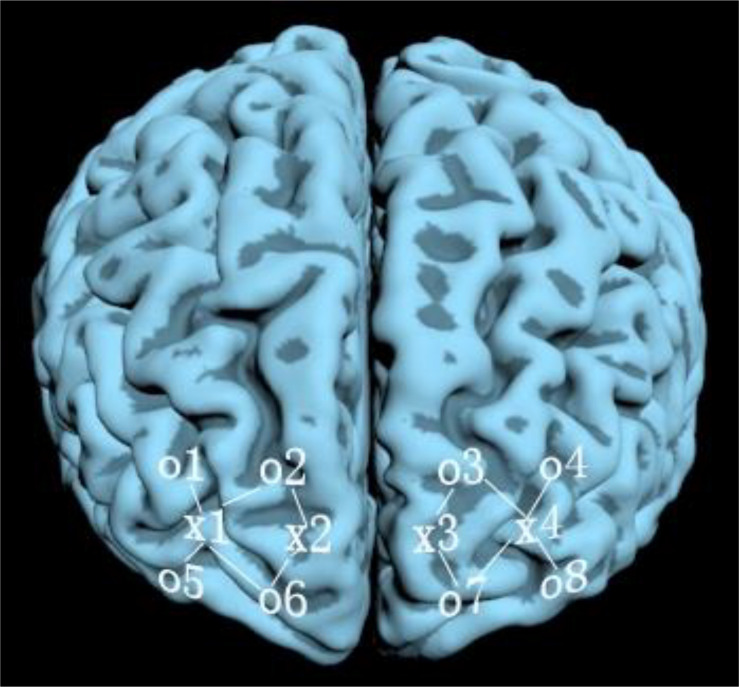
Area of prefrontal cortex map measured using fNIRS in Experiment 3. The symbol “o” before a numeral stands for a detector optode; “x” before a numeral stands for a laser optode.

**FIGURE 4:**
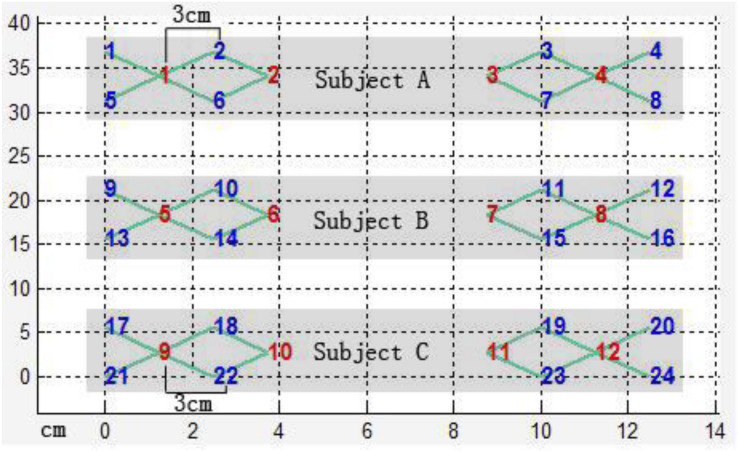
Distribution of laser optodes (red) and detectors (blue).

A TechEn CW6 continuous-wave, functional near infrared spectroscopy and imaging system was used. For the sake of efficiency in collecting the data, twelve laser optodes were connected to 24 laser sources through bifurcated cables, with one 690 nm cable and one 830 nm cable being combined into one laser optode; the optodes were evenly assigned to three participants who sat around the CW6 fNIRS equipment far apart in a triangle, watching the video simultaneously. Each child was assisted by a research assistant. The head probe consisted of three parallel strips; there were 4 laser optodes in the middle strip which were parallel to two other strips of 8 detector optodes (Figure 3). The lowest detectors were positioned along the Fp1–Fp2 line according to the international 10/20 system used in electroencephalography. The distance between each laser optode and its corresponding detector optodes was 3.0 cm. The positions of fNIRS channels are indicated in Figure 4. The sampling rate was 25 Hz.

The experimental materials were again *Tom* for the high fantasy group and *Mickey* for the low fantasy group. The Strengths and Difficulties Questionnaire (SDQ) and the battery of three behavioral EF tasks were the same as in the previous two experiments. We recorded the fNIRS of three preschoolers at a time during their viewing sessions. Each child wore a headphone and sat in front of the 17-inch non-interactive laptop screen to watch the assigned video. After the fNIRS-recorded viewing, the child was taken immediately to an adjacent quiet room to perform the EF tasks. Parents completed the SDQ while waiting for their child to complete the viewing sessions and the behavioral assessment of EF.

Hemodynamic Evoked Response (HomER) software was used to analyze Coxy-Hb linked to the oxygen inflow in brain tissue, which is a more sensitive indicator of brain activation than deoxy-Hb linked to oxygen absorption by the tissue (Hoshi et al., 2001; Strangman et al., 2002; Cui et al., 2011; Burns et al., 2018; Sun et al., 2018; Mayseless et al., 2019; Yang et al., 2020). We analyzed the Coxy-Hb of the 10 preschoolers as they watched the first 6 min and 50 s of *Tom* as well as the Coxy-Hb of the other 10 preschoolers as they watched the first segment of *Mickey* for a similar duration. In addition, we registered the total number of fantastical events and the duration of each fantastical event within each portion of stimuli that was examined.

### Results

The data from the hyperactivity subscale of the SDQ indicated that there was no significant difference in hyperactivity (*M*_high fantasy_ = 3.53, *M*_low fantasy_ = 4.00) between the two groups of preschoolers. However, the high fantasy group had significantly lower scores than the low fantasy group on the behavioral EF tasks post viewing, *t*(18) = −2.51, *p <* 0.05, Cohen’s *d* = 0.31.

**FIGURE 5:**
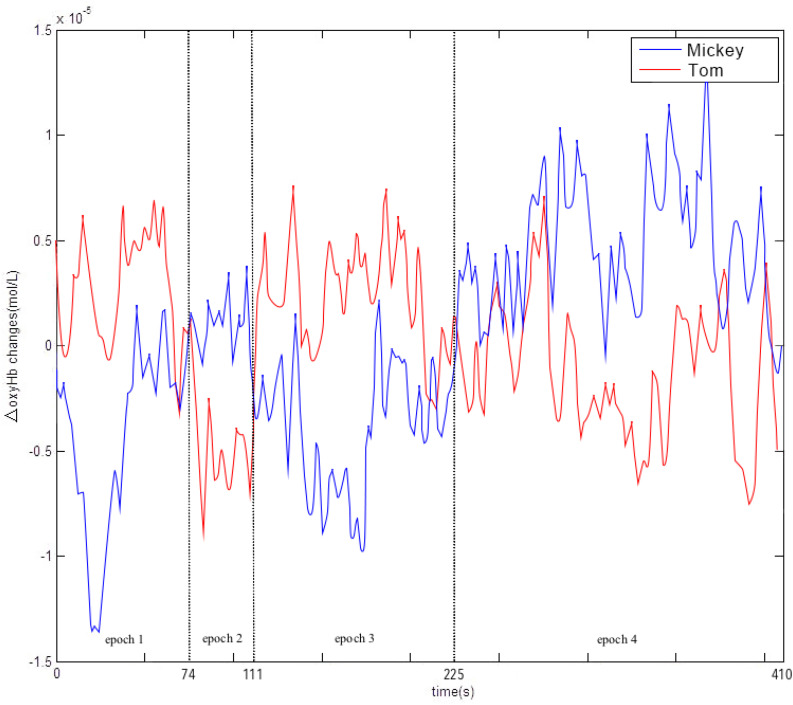
Time course for Coxy-Hb in PFC assessed by fNIRS during the first 6 min 50 s (410 s) of viewing the video with low frequency fantastical events (*Mickey*, in blue) and high frequency fantastical events (*Tom*, in red).

Visual inspection of the PFC plot suggested that the two groups were comparable overall in the level of Coxy-Hb in PFC during the 6 min 50 s viewing session (Figure 5). The curves of the two groups crossed at three time points, resulting in four epochs, in each of which one group exceeded the other to some degree in prefrontal processing. The first epoch occurred during the first 74 s of viewing. The level of prefrontal processing was significantly higher for *Tom*, the high fantasy show, *t*(19) = 2.05, *p* = 0.05, Cohen’s *d* = 0.94. The second epoch went on from 75 s to 111 s. Although in the second epoch Coxy-Hb in PFC appeared to be higher for the low fantasy group based on visual inspection, the difference was not significant, *p* > 0.05. The third epoch went on from 112 s to 225 s. There was significantly greater activation in PFC for the high fantasy group, *t*(19) = 2.32, *p* < 0.05, Cohen’s *d* = 1.06. Finally, during the last epoch from 226 s to 410 s, the group difference was not significant, *p* > 0.05. Thus, overall, there was a general trend of increasing activation in PFC from Epoch 1 to Epoch 3 in the high fantasy group.

### Discussion

The results of Experiment 3 not only replicated the findings from Experiments 1 and 2 in that the high fantasy group performed worse than the low fantasy group on EF tasks immediately after the viewing session, but also provide novel neurological evidence of disrupted EF. Specifically, Coxy-Hb in PFC was higher in the high fantasy group than the low fantasy group. Although Experiment 3 is limited by the lack of a control group, the evidence supports the hypothesis that the high fantasy group would show a higher level of Coxy-Hb, in line with Kahneman’s (1973) model of limited cognitive resources.

A close analysis of the PFC plot showed significant and remarkable differences between the high and low fantasy groups. The results of this epoch-by-epoch analysis expanded the findings of both Experiment 1 and 2, which demonstrated that the high fantasy group experienced impaired EF immediately after viewing *Tom*. These findings support earlier studies (Lillard and Peterson, 2011; Lillard et al., 2015a,b; Rhodes et al., 2020) by showing that frequent fantastical events in video programs for preschoolers consume cognitive resources and negatively impact EF. The findings also take this line of research further to show that a high level of Coxy-Hb, a measure of the use of cognitive resources, was associated with observable post-viewing EF impairment on behavioral tasks. This fNIRS evidence suggests that increases in Coxy-Hb may be a neurological mechanism of EF impairments seen in Experiments 1 and 2.

The results indicated that more fantastical events led to significantly higher activation in Epochs 1 and 3, with non-significantly different activation in Epochs 2 and 4. Consistent with Kahneman’s (1973) assertion that there is a limited capacity to process the information perceived, the results also indicate that viewing frequent fantastical events led to overloaded processing, which in turn impaired Chinese preschoolers’ EF task performance. If resources permit, it would be desirable to include a control group to measure change owing to the video treatment; so would it be ideal to assess all possible epochs in the complete viewing program. Admittedly, the current epoch analysis covered barely one third of the total length of each video. (*Tom’s* runtime is about 18.5 min and *Mickey’s* runtime about 19.5 min). A complete examination of these epochs gauged by the time code in the video stimuli could provide useful information about how viewing audiovisual fantastical events may erode preschoolers’ EF. This possibility merits future research.

## General Discussion

The three experiments in this study replicated the results of earlier research by showing that young children’s EF was weakened after watching an animated television program with frequent fantastical events. These events were defined specifically as physically impossible events as established in infant research (Woolley, 1997; Lillard and Peterson, 2011). However, our research efforts went beyond replication by introducing three new elements to this literature.

First, the samples for the three experiments were all drawn from a Chinese preschool population. Ensuing one study in China (Li et al., 2018) that compared the different effects of TV and the touch screen on preschoolers’ EF, the current study is the first to extend this line of research from the United States and the United Kingdom into the Chinese preschool population. Chinese preschoolers have been shown to have higher inhibition and attention control (Lan et al., 2011) and higher scores on EF measures (Sabbagh et al., 2006; Tan, 2020) than United States preschoolers; however, in the current three experiments, Chinese preschoolers like their counterparts in the other two countries faced excessive demands on cognitive resources associated with watching frequent fantastical events on TV. Most likely, preschoolers in all three countries encountered the same cognitive and behavioral challenges in completing the EF tasks, which demanded cognitive resources like working memory, inhibition of action and flexible thinking. In this regard, in addition to Kahneman’s (1973) and Fisch’s (2000) model, Lang’s (2000) model also explains that, while viewing television, a preschooler is an active information processor and at the same time has a limited capacity to process the information perceived. “When a viewer has insufficient resources available to perform all of these subprocesses thoroughly, some aspects of processing will suffer” (Lang, 2000, p. 55). The Chinese preschoolers appeared to experience the cognitive resource insufficiency.

Second, the use of eye tracking technology in the second experiment provided a different type of evidence of disrupted EF than was available in previous research. This psychophysiological evidence showed that preschoolers’ frequent and short eye fixations may require greater mobilization or even maximization of their limited processing capacity. At the same time, the eye fixation data in Experiment 2 are indicative of the impact of frequent fantastical events on the young viewer’s psychophysical resources as their attention shifted, likely pushing to the edge of preschoolers’ processing resource capacity as Kahneman (1973) suggested. The eye tracking data from Experiment 2 provided new evidence parallel to the findings from Experiment 1 and other studies, and strongly suggested that preschoolers’ psychophysiological resources were overdrawn. This finding suggests a plausible explanation for the weakened EF performance immediately after viewing frequent fantastic events on the screen.

Third, if the second experiment offered psychophysiological evidence of disrupted EF, the third experiment in turn offered neurobehavioral evidence by using fNIRS to measure the likely use of cognitive resources. This method allowed us to directly measure Coxy-Hb in brain tissues as an indicator of PFC activation, or as Petrides (2000) put it, to engage in a necessary higher level of monitoring or processing the audiovisual task. These results by far offer a more direct measure of observable neural activity while preschoolers watch fantastic events on the screen. The findings corroborate others’ results (Lillard and Peterson, 2011; Lillard et al., 2015a,b; Rhodes et al., 2020) and those of our first and second experiments, and lend further support for the conceptual model of limited resource capacity. More importantly, they suggest an underlying mechanism that would explain disruptions in preschoolers’ EF after processing fantastic events.

The key limitation of this study concerns differences between the two stimulus videos; these differences might have introduced confounds and call for caution against drawing strong conclusions. The first is the video selection. Practically, it would be almost impossible to find animation programs that are similar in all respects except the frequency of fantastical events, a problem that can be significantly alleviated by a media production team’s support in creating novel story-based animations. We focused mainly on identifying videos that included a noticeably different number of fantastical events and were rated by experts as developmentally appropriate for preschool children (Common Sense Media, 2020; Sheppard, 2020). However, *Mickey* presented one continuous story without any conflicts while *Tom* presented three shorter episodes with a few comedic characters involved in animated conflicts. This one-to-three-episode arrangement was to match the total viewing durations, but it gave rise to an incompatibility that *Mickey* provides one narrative, “the story presented in the program” (Fisch, 2000, p. 64), whereas *Tom* presents three action-based “episodic narratives” (Hilmes et al., 2014, p. 27). It may be argued that, in this arrangement, *Tom* would be more difficult to understand because it might demand more mental resources, thus supporting the explanation that added narratives might weaken the *Tom* viewing group’s EF.

However, there is another critical underlying difference: *Mickey* is by default an educational program and *Tom* an entertaining one. If we follow Fisch’s (2000) model on how children use their resources to understand the educational content on TV, *Mickey* may not present preschoolers with fewer resource draining events than *Tom* because, in addition to processing the narrative in *Mickey*, its viewers also need to process the educational content and the narrative that carries such content. In other words, this comparison shows a small tip of a large set of complex issues embedded in the video stimuli in the study. *Tom* in the three short episodes may not present preschoolers with much more complexity for processing than *Mickey* in one story, but *Tom* may create greater disruption in the viewer’s sense of continuity and in eye orienting responses, both of which may affect EF. Although the current study did not address this issue, it points to a worthwhile research direction in comparing the in-depth difference between educational and entertaining program content on TV, using Fisch’s (2000) limited capacity model.

Another critical issue is whether fantastical events could be differentiated from the so-called comedic violence as a criterion for distinguishing *Mickey* from *Tom.* A close observation of fantastical event scenes from the two videos suggests that impossible events violate most children’s daily experiences. For example, when two actors in *Tom* pull open a sandwich in which Jerry hides, Jerry hangs on between the split sandwich pieces. Both of his arms are stretched a few times longer than his body. On the other hand, Mickey walks in a circle when his tail suddenly coils up like a spring and he starts bouncing on it as a way of walking. A sharp difference between the two scenes is that one occurs under an external force and the other out of an instant magic, but a clear commonality is that they both show an impossible bodily distortion with an incredible function. A small number of studies have shown that laboratory experiments consistently fail to reveal a positive correlation between viewing comedic violence and aggressive behaviors in early childhood (see Kirsh, 2006, for a review). This failure adds a layer of uncertainty about how the Chinese preschoolers perceived those comedic scenes of violence in *Tom* and whether viewing these scenes would impact EF. In short, there remains a gap in the literature regarding video content and preschoolers’ perception thereof. The frequency of fantastic events appears to be a definable and operational gauge of the differences between the two study videos that might have affected preschoolers’ EF immediately after the viewing session. This study is an initial step toward examining the impact of fantastical events in animation on Chinese preschoolers’ EF, with methods that are new to the area of research.

Another possible confound was the animation technique used to create each video. Did the traditional animation technique in *Tom* affect preschoolers’ EF more negatively than the computer animation in *Mickey*? It is little known whether traditional animation plays a different role from computer animation in children’s viewing experience and learning, less so in their EF. In other fields, researchers have noted that young children prefer to imitate and learn from a live model or a teacher rather than a mechanical model or a robot (e.g., Meltzoff, 1995; Kuhl et al., 2003; Moriguchi et al., 2011; Moriguchi and Hiraki, 2014). A similar line of research is worth pursuing to detect children’s preferences for traditional or computer animation technology.

There is a caveat about studies on the effects of television on children’s attention, which is part of EF. Although more than 70% of these studies found a negative association between television viewing and children’s attention quality, such evidence may be inadequate without placing children in their social context and taking into account other factors that could affect attention (Kostyrka-Allchorne et al., 2017). Close-to-real-life fantastical contents or contextualized fantasy in a TV program can facilitate preschoolers’ performance on cognitive tasks (Richert and Schlesinger, 2016).

Despite the limitations, we believe the study has heuristic value for further research on the link between the cognitive demands of processing fantastical events and the immediate effect of these demands on subsequent EF. The implications of our findings for future research remain to be further explored. For example, how long will the immediate negative effect of fantastical events on EF last? Bonus and Mares (2015) suggest that, as time goes by, children become more and more skeptical about the authenticity of fantasy content in TV. So, a reasonable question is: Can the negative effect become stronger or weaker over time? A developmental perspective, a large sample, and a pair of robust stimuli could allow us to explore the possible developmental EF outcomes resulting from preschoolers’ viewing of fantastical events. For example, video clips with well-defined fantastical events could be used to compare preschool viewers’ EF one hour, two hours or four hours after viewing. It would be also helpful to combine eye tracking and fNIRS in one experimental procedure to determine if there are coordinated patterns of association between eye movement and Coxy-Hb in PFC. In short, both the conceptual model and the technology we used in this study, along with our findings, suggest a set of new future experiments.

In conclusion, the findings contribute to the current body of literature through a series of three experiments using behavioral, psychophysiological and neurobehavior evidence. They consistently show that preschoolers experience EF disruption immediately after viewing fantastical events. The results across experiments provide support for Kahneman’s and other theorists’ models of limited processing capacity as an explanation for disruptions in EF after watching fantastical events. The current study also makes several contributions to the literature. This is the first study on this topic that has been conducted in China and it was also the first study to use eye tracking and fNIRS to document the psychophysiological and neural underpinnings of preschoolers’ disrupted EF after watching animated fantastical events. In addition, this study is the first to conceptualize these EF disruptions in terms of the theoretical tradition of limited cognitive capacity. Although there is reason to be cautious in interpreting the results, the findings extend and enrich the early behavioral evidence of the immediate negative effects of “incomprehensible events” on preschoolers’ EF.

## Data Availability Statement

The raw data supporting the conclusions of this article will be made available by the authors, without undue reservation.

## Ethics Statement

The Human Research Ethics Committee at Central China Normal University approved the experiment’s materials and procedures. Parents provided written informed consent for their child to participate in the study.

## Author Contributions

HL developed the study concept. YH and HL were primarily responsible for writing the manuscript, with all remaining authors providing critical revisions. All authors approved the final version of the manuscript for submission.

## Conflict of Interest

The authors declare that the research was conducted in the absence of any commercial or financial relationships that could be construed as a potential conflict of interest.

## References

[B1] Abraham A. Von Cramon D. Y. Schubotz R. I. (2008). Meeting George Bush versus meeting Cinderella: the neural response when telling apart what is real from what is fictional in the context of our reality. *J. Cogn. Neurosci.* 20 965–976. 10.1162/jocn.2008.20059 18211244

[B2] American Academy of Pediatrics (2016). *American Academy of Pediatrics Announces New Recommendations for Children’s Media Use.* Available at: https://www.aap.org/en-us/about-the-aap/aap-press-room/Pages/AmericanAcademy-of-Pediatrics-Announces-New-Recommendations-for-ChildrensMedia-Use.aspx [Accessed June 15, 2018]

[B3] Anderson D. R. Davidson M. C. (2019). Receptive versus interactive video screens: a role for the brain’s default mode network in learning from media. *Comp. Hum. Behav.* 99 168–180. 10.1016/j.chb.2019.05.008

[B4] Anderson D. R. Hanson K. G. (2009). Children, media, and methodology. *Am. Behav. Sci.* 52 1204–1219. 10.1177/0002764209331542

[B5] Barr R. Lauricella A. Zack E. Calvert S. L. (2010). Infant and early childhood exposure to adult-directed and child-directed television programming: relations with cognitive skills at age four. *Merrill Palmer Q.* 56 21–48. 10.1353/mpq.0.0038

[B6] Bascandziev I. Powell L. J. Harris P. L. Carey S. (2016). A role for executive functions in explanatory understanding of the physical world. *Cogn. Dev.* 39 71–85. 10.1016/j.cogdev.2016.04.001

[B7] Blair C. Razza R. P. (2007). Relating effortful control, executive function, and false belief understanding to emerging math and literacy ability in kindergarten. *Child Dev.* 78 647–663. 10.1111/j.1467-8624.2007.01019.x 17381795

[B8] Bonus J. A. (2019). The impact of pictorial realism in educational science television on US children’s learning and transfer of biological facts. *J. Child. Media* 13 433–451. 10.1080/17482798.2019.1646295

[B9] Bonus J. A. Mares M. L. (2015). Learned and remembered but rejected: preschoolers’ reality judgments and transfer from Sesame Street. *Commun. Res.* 46 375–400. 10.1177/0093650215609980

[B10] Bunce L. Palmer S. Lockley S. Boerger E. Woolley J. (2017). Imagining the impossible and the development of creative abilities. *Paper presented at the Society for Research in Child Development (SRCD)*, Austin, TX.

[B11] Burns S. M. Barnes L. N. Katzman P. L. Ames D. L. Falk E. B. Lieberman M. D. (2018). A functional near infrared spectroscopy (fNIRS) replication of the sunscreen persuasion paradigm. *Soc. Cogn. Affect. Neurosci.* 13 628–636. 10.1093/scan/nsy030 29733408 PMC6022533

[B12] Carlson S. M. Zelazo P. D. Faja S. (2012). “Executive function,” in *Oxford handbook of Developmental Psychology*, ed. Zelazo P. D. (New York, NY: Oxford University Press), 706–743.

[B13] Cheng S. Maeda T. Yoichi S. Yamagata Z. Tomiwa K. Japan Children’s Study Group. (2010). Early television exposure and children’s behavioral and social outcomes at age 30 months. *J. Epidemiol.* 20(Suppl. 2) S482–S489. 10.2188/jea.je20090179 20179364 PMC3920399

[B14] Common Sense Media (2017). *The Common Sense Census: Media Use by Kids Age Zero to Eight 2017.* San Francisco, CA: Common Sense Media.

[B15] Common Sense Media (2020). *Mickey Mouse Clubhouse.* San Francisco, CA: Common Sense Media.

[B16] Conners-Burrow N. A. McKelvey L. Fussell J. J. (2011). Social outcomes associated with media viewing habits of low-income preschool children. *Early Educ. Dev.* 22 256–273. 10.1080/10409289.2011.550844

[B17] Cooper N. R. Uller C. Pettifer J. Stolc F. C. (2009). Conditioning attentional skills: examining the effects of the pace of television editing on children’s attention. *Acta Paediatr.* 98 1651–1655. 10.1111/j.1651-2227.2009.01377.x 19500080

[B18] Cui X. Bray S. Bryant D. M. Glover G. H. Reiss A. L. (2011). A quantitative comparison of NIRS and fMRI across multiple cognitive tasks. *NeuroImage* 54 2808–2821. 10.1016/j.neuroimage.2010.10.069 21047559 PMC3021967

[B19] d’Ydewalle G. De Bruycker W. (2007). Eye movements of children and adults while reading television subtitles. *Eur. Psychol.* 12 196–205. 10.1027/1016-9040.12.3.196

[B20] Ebenegger V. Marques-Vidal P. M. Munsch S. Quartier V. Nydegger A. Barral J. (2012). Relationship of hyperactivity/inattention with adiposity and lifestyle characteristics in preschool children. *J. Child Neurol.* 27 852–858. 10.1177/0883073811428009 22209757

[B21] Eisenberg N. Smith C. L. Sadovsky A. Spinrad T. L. (2004). “Effortful control: relations with emotion regulation, adjustment, and socialization in childhood,” in *Handbook of Self-Regulation: Research, Theory, and Applications*, eds Baumeister R. F. Vohs K. D. (New York, NY: Guilford), 259–282.

[B22] Evans M. A. Saint-Aubin J. (2005). What children are looking at during shared storybook reading. *Psychol. Sci.* 16 913–920. 10.1111/j.1467-9280.2005.01636.x 16262779

[B23] Fisch S. M. (2000). A capacity model of children’s comprehension of educational content on television. *Media Psychol.* 2 63–91. 10.1207/S1532785XMEP0201_4

[B24] Foster E. M. Watkins S. (2010). The value of reanalysis: TV viewing and attention problems. *Child Dev.* 81 368–375. 10.1111/j.1467-8624.2009.01400.x 20331673

[B25] Frank M. C. Amso D. Johnson S. P. (2014). Visual search and attention to faces during early infancy. *J. Exp. Child Psychol.* 118 13–26. 10.1016/j.jecp.2013.08.012 24211654 PMC3844087

[B26] Frick A. Mohring W. Newcombe N. (2014). Development of mental transformation abilities. *Trends Cogn. Sci.* 18 536–542. 10.1016/j.tics.2014.05.011 24973167

[B27] Funahashi S. (2001). Neuronal mechanisms of executive control by the prefrontal cortex. *Neurosci. Res.* 39 147–165. 10.1016/S0168-0102(00)00224-8 11223461

[B28] Gannaway B. (2007). *Mickey’s Color Adventure [DVD].* California, CL: Disney Television Animation (Translated into Chinese and published by Excel Media/Guandong Co. Ltd., March, 2009.).

[B29] Garon N. Bryson S. E. Smith I. M. (2008). Executive function in preschoolers: a review using an integrative framework. *Psychol. Bull.* 134 31–60. 10.1037/0033-2909.134.1.31 18193994

[B30] Geist E. A. Gibson M. (2000). The effect of network and public television programs on four and five year olds’ ability to attend to educational tasks. *J. Instr. Psychol.* 27 250–250.

[B31] Gerstadt C. L. Hong Y. J. Diamond A. (1994). The relationship between cognition and action: performance of children 3(1/2)-7 years old on a stroop-like day-night test. *Cognition* 53 129–153. 10.1016/0010-0277(94)90068-X 7805351

[B32] Goodman R. (2001). Psychometric properties of the Strengths and Difficulties Questionnaire. *J. Am. Acad. Child Adolesc. Psychiatry* 40 1337–1345. 10.1097/00004583-200111000-00015 11699809

[B33] Goswami U. (2008). *Cognitive Development: The learning Brain.* London: Psychology Press.

[B34] Gredebäck G. Johnson S. Von Hofsten C. (2010). Eye tracking in infancy research. *Dev. Neuropsychol.* 35 1–19. 10.1080/87565640903325758 20390589

[B35] Hawkins R. P. Pingree S. Bruce L. Tapper J. (1997). Strategy and style in attention to television. *J. Broadcast. Electron. Media* 41 245–264. 10.1080/08838159709364404

[B36] Hilmes M. Huber C. Jaramillo D. Martin A. Milch D. Nayman A. (2014). Rethinking television: a critical symposium on the new age of episodic narrative storytelling. *Cineaste* 39 26–38.

[B37] Hoshi Y. Kobayashi N. Tamura M. (2001). Interpretation of near-infrared spectroscopy signals: a study with a newly developed perfused rat brain model. *J. Appl. Physiol.* 90 1657–1662. 10.1152/jappl.2001.90.5.1657 11299252

[B38] Hsin C. Li M. Tsai C. (2014). The influence of young children’s use of technology on their learning: a review. *J. Educ. Technol. Soc.* 17 85–99.

[B39] Hyönä J. (2010). The use of eye movements in the study of multimedia learning. *Learn. Instr.* 20 172–176. 10.1016/j.learninstruc.2009.02.013

[B40] Jacques S. Zelazo P. D. (2001). The flexible item selection task (FIST): a measure of executive function in preschoolers. *Dev. Neuropsychol.* 20 573–591. 10.1207/S15326942DN2003_2 12002094

[B41] Jones C. Noble M. Maltese M. (1964). *The Cat above and the Mouse Below [DVD].* California, CL: Metro-Goldwyn-Mayer.

[B42] Kahneman D. (1973). *Attention and Effort.* Englewood Cliffs, NJ: Prentice Hall.

[B43] Kirkorian H. L. Anderson D. R. Keen R. (2012). Age differences in online processing of video: an eye movement study. *Child Dev.* 83 497–597. 10.1111/j.1467-8624.2011.01719.x 22288510 PMC3305831

[B44] Kirsh S. J. (2006). Cartoon violence and aggression in youth. *Aggress. Violent Behav.* 11 547–557. 10.1016/j.avb.2005.10.002

[B45] Kolling T. Óturai G. Knopf M. (2014). Is selective attention the basis for selective imitation in infants? An eye-tracking study of deferred imitation with 12-month-olds. *J. Exp. Child Psychol.* 124 18–35. 10.1016/j.jecp.2014.01.016 24727296

[B46] Kostyrka-Allchorne K. Cooper N. R. Simpson A. (2017). The relationship between television exposure and children’s cognition and behaviour: a systematic review. *Dev. Rev.* 44 19–58. 10.1016/j.dr.2016.12.002

[B47] Kuhl P. K. Tsao F. M. Liu H. M. (2003). Foreign-language experience in infancy: effects of short-term exposure and social interaction on phonetic learning. *Proc. Nat. Acad. Sci. U.S.A.* 100 9096–9101. 10.1073/pnas.1532872100 12861072 PMC166444

[B48] Lan X. Legare C. H. Ponitz C. C. Li S. Morrison F. J. (2011). Investigating the links between the subcomponents of executive function and academic achievement: a cross-cultural analysis of Chinese and American preschoolers. *J. Exp. Child Psychol.* 108 677–692. 10.1016/j.jecp.2010.11.001 21238977

[B49] Lang A. (2000). The limited capacity model of mediated message processing. *J. Commun.* 50 46–70. 10.1111/j.1460-2466.2000.tb02833.x

[B50] Lewis C. Koyasu M. Oh S. Ogawa A. Short B. Huang Z. (2009). Culture, executive function, and social understanding. *New Dir. Child Adolesc. Dev.* 123 69–85. 10.1002/cd.236 19306275

[B51] Li H. Boguszewski K. Lillard A. S. (2015). Can that really happen? Children’s knowledge about the reality status of fantastical events in television. *J. Exp. Child Psychol.* 139 99–114. 10.1016/j.jecp.2015.05.007 26094241

[B52] Li H. Liu T. Woolley J. D. Zhang P. (2019). Reality status judgments of real and fantastical events in children’s prefrontal cortex: an fNIRS study. *Front. Hum. Neurosci.* 13:444. 10.3389/fnhum.2019.00444 31992977 PMC6933013

[B53] Li H. Subrahmanyam K. Bai X. Xie X. Liu T. (2018). Viewing fantastical events versus touching fantastical events: short-term effects on children’s inhibitory control. *Child Dev.* 89 48–57. 10.1111/cdev.12820 28478648

[B54] Lillard A. S. Drell M. B. Richey E. M. Boguszewski K. Smith E. D. (2015a). Further examination of the immediate impact of television on children’s executive function. *Dev. Psychol.* 51 792–805. 10.1037/a0039097 25822897

[B55] Lillard A. S. Li H. Boguszewski K. (2015b). Television and children’s executive function. *Adv. Child Dev. Behav.* 48 219–249. 10.1016/bs.acdb.2014.11.006 25735946

[B56] Lillard A. S. Peterson J. (2011). The immediate impact of different types of television on young children’s executive function. *Pediatrics* 128 644–649. 10.1542/peds.2010-1919 21911349 PMC9923845

[B57] Linebarger D. L. Barr R. Lapierre M. A. Piotrowski J. T. (2014). Associations between parenting, media use, cumulative risk, and children’s executive functioning. *J. Dev. Behav. Pediatr.* 35 367–377. 10.1097/DBP.0000000000000069 25007059

[B58] Mares M. L. Sivakumar G. (2014). “Vámonos means go, but that’s made up for the show”: reality confusions and learning from educational TV. *Dev. Psychol.* 50 2498–2511. 10.1037/a0038041 25347304

[B59] Martin A. Razza R. A. Brooks-Gunn J. (2012). Specifying the links between household chaos and preschool children’s development. *Early Child Dev. Care* 182 1247–1263. 10.1080/03004430.2011.605522 22919120 PMC3422884

[B60] Mayseless N. Hawthorne G. Reiss A. L. (2019). Real-life creative problem solving in teams: fNIRS based hyperscanning study. *NeuroImage* 203 116–161. 10.1016/j.neuroimage.2019.116161 31493532

[B61] McEwen R. N. Dubé A. K. (2015). Engaging or distracting: Children’s tablet computer use in education. *J. Educ. Technol. Soc.* 18 9–23.

[B62] Meltzoff A. N. (1995). Understanding the intentions of others: re-enactment of intended acts by 18-month-old children. *Dev. Psychol.* 31 838–850. 10.1037/0012-1649.31.5.838 25147406 PMC4137788

[B63] Miller C. J. Marks D. J. Miller S. R. Berwid O. G. Kera E. C. Santra A. (2007). Brief report: television viewing and risk for attention problems in preschool children. *J. Pediatr. Psychol.* 32 448–452. 10.1093/jpepsy/jsl035 17012738

[B64] Mischel W. Shoda Y. Rodriguez M. I. (1989). Delay of gratification in children. *Science* 244 933–938. 10.1126/science.2658056 2658056

[B65] Moffitt T. E. Arseneault L. Belsky D. Dickson N. Hancox R. J. Harrington H. (2011). A gradient of childhood self-control predicts health, wealth, and public safety. *Proc. Natl. Acad. Sci.U.S.A.* 108 2693–2698. 10.1073/pnas.1010076108 21262822 PMC3041102

[B66] Moriguchi K. Hiraki K. Mishkin M. (2009). Neural origin of cognitive shifting in young children. *Proc. Natl. Acad. Sci. U.S.A.* 106 6017–6021. 10.1073/pnas.0809747106 19332783 PMC2667026

[B67] Moriguchi Y. Hiraki K. (2011). Longitudinal development of prefrontal function during early childhood. *Dev. Cogn. Neurosci.* 1 153–162. 10.1016/j.dcn.2010.12.004 22436437 PMC6987577

[B68] Moriguchi Y. Hiraki K. (2014). Neural basis of learning from television in young children. *Trends Neurosci. Educ.* 3 122–127. 10.1016/j.tine.2014.07.001

[B69] Moriguchi Y. Kanda T. Ishiguro H. Shimada Y. Itakura S. (2011). Can young children learn words from a robot? *Interact. Stud.* 12 107–118. 10.1075/is.12.1.04mor

[B70] Morita T. Slaughter V. Katayama N. Kitazaki M. Kakigi R. Itakura S. (2012). Infant and adult perceptions of possible and impossible body movements: an eye tracking study. *J. Exp. Child Psychol.* 113 401–414. 10.1016/j.jecp.2012.07.003 22906302

[B71] Nathanson A. I. Aladé F. Sharp M. L. Rasmussen E. E. Christy K. (2014). The relation between television exposure and executive function among preschoolers. *Dev. Psychol.* 50 1497–1506. 10.1037/a0035714 24447117

[B72] Papavlasopoulou S. Sharma K. Giannakos M. N. (2018). How do you feel about learning to code? Investigating the effect of children’s attitudes towards coding using eye-tracking. *Int. J. Child Comput. Interact.* 17 50–60. 10.1016/j.ijcci.2018.01.004

[B73] Petrides M. (2000). Dissociable roles of mid-dorsolateral prefrontal and anterior inferotemporal cortex in visual working memory. *J. Neurosci.* 20 7496–7503. 10.1523/JNEUROSCI.20-19-07496.2000 11007909 PMC6772785

[B74] Przybylski A. K. Weinstein N. (2019). Digital screen time limits and young children’s psychological well-being: evidence from a population-based study. *Child Dev.* 90 56–65. 10.1111/cdev.13007 29235663

[B75] Quimby F. Barbera J. Hanna W. (1949). *Jerry’s Diary [DVD].* California, CL: Metro-Goldwyn-Mayer.

[B76] Quimby F. Hanna W. Barbera J. (1945). *Flirty Birdy [DVD].* California, CL: Metro-Goldwyn-Mayer.

[B77] Rhodes S. M. Stewart T. M. Kanevski M. (2020). Immediate impact of fantastical television content on children’s executive functions. *Br. J. Dev. Psychol.* 38 268–288. 10.1111/bjdp.12318 31872905

[B78] Richert R. A. Schlesinger M. A. (2016). The role of fantasy-reality distinctions in preschoolers’ learning from educational video. *Infant Child Dev.* 26:e2009 10.1002/icd.2009

[B79] Sabbagh M. A. Xu F. Carlson S. M. Moses L. J. Lee K. (2006). The development of executive functioning and theory of mind a comparison of Chinese and US preschoolers. *Psychol. Sci.* 17 74–81. 10.1111/j.1467-9280.2005.01667.x 16371147 PMC2567057

[B80] Sheppard D. (2020). *Tom and Jerry: Common Sense Media TV Review.* Available at: https://www.commonsensemedia.org/tv-reviews/tom-and-jerry [Accessed March 16, 2020]

[B81] Shtulman A. Carey S. (2007). Improbable or impossible? How children reason about the possibility of extraordinary events. *Child Dev.* 78 1015–1032. 10.1111/j.1467-8624.2007.01047.x 17517019

[B82] Spelke E. (1994). Initial knowledge: six suggestions. *Cognition* 50 431–445. 10.1016/0010-0277(94)90039-6 8039373

[B83] Stevens T. Mulsow M. (2006). There is no meaningful relationship between television exposure and symptoms of attention-deficit/hyperactivity disorder. *Pediatrics* 117 665–672. 10.1542/peds.2005-0863 16510645

[B84] Strangman G. Culver J. P. Thompson J. H. Boas D. A. (2002). A quantitative comparison of simultaneous BOLD fMRI and NIRS recordings during functional brain activation. *Neuroimage* 17 719–731. 10.1006/nimg.2002.1227 12377147

[B85] Subbotsky E. Hysted C. Jones N. (2010). Watching films with magical content facilitates creativity in children. *Percept. Mot. Skills* 111 261–277. 10.2466/04.09.11.pms.111.4.261-277 21058605

[B86] Sun P. P. Tan F. L. Zhang Z. Jiang Y. H. Zhao Y. Zhu C. Z. (2018). Feasibility of functional near-infrared spectroscopy (fNIRS) to investigate the mirror neuron system: an experimental study in a real-life situation. *Front. Hum. Neurosci.* 12:86. 10.3389/fnhum.2018.00086 29556185 PMC5845015

[B87] Tan B. (2020). *Chinese and U.S. Young Children’s Executive Function and its Socialiocultural Antecedents*, Dissertation/master’s thesis, University of Memphis, Memphis, TN.

[B88] Thibodeau-Nielsen R. B. Gilpin A. T. Brown M. M. Meyer B. A. (2016). The effects of fantastical pretend-play on the development of executive functions: an intervention study. *J. Exp. Child Psychol.* 145 120–138. 10.1016/j.jecp.2016.01.001 26835841

[B89] Thibodeau-Nielsen R. B. Gilpin A. T. Nancarrow A. F. Pierucci J. M. Brown M. M. (2020). Fantastical pretense’s effects on executive function in a diverse sample of preschoolers. *J. Appl. Dev. Psychol.* 68 101–137. 10.1016/j.appdev.2020.101137

[B90] Tsujimoto S. Yamamoto T. Kawaguchi H. Koizumi H. Sawaguchi T. (2004). Prefrontal cortical activation associated with working memory in adults and preschool children: an event-related optical topography study. *Cereb. Cortex* 14 703–712. 10.1093/cercor/bhh030 15084489

[B91] Tu Y. (2009). Viewing the development of Chinese animation from the invasion of foreign animation. *Charm. China* 07:146.

[B92] Verlinden M. Tiemeier H. Hudziak J. J. Jaddoe V. W. Raat H. Guxens M. (2012). Television viewing and externalizing problems in preschool children: the generation R study. *Arch. Pediatr. Adolesc. Med.* 166 919–925. 10.1001/archpediatrics.2012.653 22869354

[B93] Wass S. V. (2016). “The use of eye tracking with infants and children,” in *Practical Research with Children*, eds Prior J. van Henwegen J. (New York, NY: Routledge), 24–45.

[B94] Woolley J. D. (1997). Thinking about fantasy: are children fundamentally different thinkers and believers from adults? *Child Dev.* 68 991–1011. 10.1111/j.1467-8624.1997.tb01975.x 9418217

[B95] Wright J. C. Huston A. C. Ross R. P. Calvert S. L. Rolandelli D. Weeks L. A. (1984). Pace and continuity of television programs: effects on children’s attention and comprehension. *Dev. Psychol.* 20 653–666. 10.1037/0012-1649.20.4.653

[B96] Wu C. J. Liu C. Y. Yang C. H. Jian Y. C. (2020). Eye-movements reveal children’s deliberative thinking and predict performance on arithmetic word problems. *Eur. Eur. J. Psychol. Educ.* 10.1007/s10212-020-00461-w [Epub ahead of print].

[B97] Yang J. Zhang H. Ni J. Dreu C. K. W. D. Ma Y. (2020). Within-group synchronization in the prefrontal cortex associates with intergroup conflict. *Nat. Neurosci.* 23 754–760. 10.1038/s41593-020-0630-x 32341541

[B98] Yang X. H. Chen Z. Wang Z. H. Zhu L. Q. (2017). The relations between television exposure and executive function in Chinese preschoolers: the moderated role of parental mediation behaviors. *Front. Psychol.* 8:1833. 10.3389/fpsyg.2017.01833 29089912 PMC5651076

